# The phenomenology and natural history of idiopathic lower cranial dystonia

**DOI:** 10.1186/2054-7072-1-3

**Published:** 2014-10-29

**Authors:** Pichet Termsarasab, Donald R Tanenbaum, Steven J Frucht

**Affiliations:** Department of Neurology, Movement Disorders Division, Icahn School of Medicine at Mount Sinai, 5 East 98th St, first floor, New York, NY 10029 USA

**Keywords:** Lower cranial dystonia, Oromandibular dystonia, Geste antagoniste

## Abstract

**Background:**

Many patients with lower cranial dystonia (LCrD) are misdiagnosed, and recognition of this condition by general practitioners and dental health professionals is limited.

**Methods:**

We define the phenomenology and natural history of idiopathic LCrD, presenting in 41 patients with the disorder, the largest series of these patients reported to date.

**Results:**

Phenomenology of dystonia included lower cranial and pharyngeal involvement, jaw opening and jaw closing dystonia, and tongue dystonia. Of 25 newly described patients, 72% (18) were female, average age at onset was 56 years, and delay before correct diagnosis was 3.8 years (0-25 years, median 2 years). Eleven patients (44%) reported a precipitating event, the most common of which was recent dental work. Geste antagonistes were found in 18 patients (72%). Response to treatment was mixed, indicating an unmet therapeutic need.

**Conclusions:**

Idiopathic LCrD is often missed and institution of effective therapy is often delayed. The clinical features and natural history of LCrD are similar to other forms of focal dystonia.

**Electronic supplementary material:**

The online version of this article (doi:10.1186/2054-7072-1-3) contains supplementary material, which is available to authorized users.

## Background

Lower cranial dystonia (LCrD), focal dystonia of the muscles of the lower face, jaw, tongue and pharynx, may be seen in a variety of scenarios including tardive dystonia, DYT6 and DYT12, and in neurodegenerative disorders such as Wilson’s disease and neurodegeneration with brain iron accumulation (NBIA). In contrast, isolated LCrD occurring without other abnormalities is distinctly rare. Many patients with LCrD are misdiagnosed, and recognition of this condition by general practitioners and dental health professionals is limited, delaying appropriate diagnosis and treatment. Two hundred and twenty eight patients with lower cranial dystonia have been reported in at least 8 prior case series/reports [1–8], however significant limitations in these reports include; 1) mixing of patients with tardive or other secondary forms of LCrD; 2) inclusion of patients with dystonia in other body regions such as the upper face or neck; and, 3) lack of detailed phenomenologic descriptions. We present 41 patients with idiopathic LCrD (25 newly reported), the largest report to date of idiopathic LCrD, in order to increase awareness and facilitate diagnosis and treatment of this unusual condition.

## Methods

Twenty-five patients with idiopathic LCrD were evaluated by the senior movement disorders neurologist (SJF) over a period of eight years. Many patients were referred by dentists or oral surgeons (DT). Patients with secondary dystonia or with exposure to dopamine receptor blocking agents were excluded. Clinical history, examination, and video review were performed (PT, SJF). Age at onset, duration of symptoms, precipitating factors, clinical phenomenology, the presence or absence of geste maneuvers, task-specificity, aggravating and relieving factors, and associated symptoms and treatment response are summarized in Table 1.

**Table 1:** **Summary of clinical features including phenomenology of 41 patients with idiopathic LCrD**

Number	Gender	Age at onset	Duration	Areas affected	Primary movement	Task specificity	Precipitating event	Sensory trick	Associated symptoms	Treatment	Oral device; response
1	F	65	2	Jaw	Jaw opening	Speech, eating, chewing	Dental work	None	None	LD (no), BoNT (no, dysphagia), THP (no), CLZ (no), baclofen (unknown)	N
2	F	71	1	Jaw	L jaw deviation	Eating, chewing, speech	Mandibular fx after a fall	None	None	THP (yes, 2–4 mg/d)	N
3	F	59	2	Jaw	Jaw opening and protrusion	Speech -- > eating, chewing	Maxillary bridge replacement	Plastic between teeth	Pain/clicking in TMJ	THP (no)	N
4	F	53	0	Jaw	Jaw opening	Speech -- > eating	None	Plastic/tongue depressor between teeth > holding jaw	R ear clicking, lower jaw pain	THP (no), BoNT (no), Baclofen (no), TBZ (no, depression), CLZ (no, sedation), DZP (unknown)	N
5	F	46	2	Jaw	Jaw opening	Speech -- > eating	None	Plastic between teeth, holding jaw	None	CLZ (no), THP (unknown), BoNT (yes, mild)	Y; initial response
6	M	61	1	Pharynx, larynx	Pharyngeal/laryngeal dystonia	Speech (worse with ke, ge sounds)	R sinus lift and dental implant	Plastic/tongue depressor between teeth	None	THP (yes, 6–15 mg/d)	N
7	F	73	1	Jaw	R jaw deviation	At rest	None	Plastic between teeth on the L	None	THP (no), CLZ (1 mg/d, unknown)	N
8	F	70	1	Jaw	Jaw opening	Speech	Cheering a football game	Tongue depressor between lip/teeth; holding jaw	None	CLZ (no), LD (no), THP (no), Baclofen (no), BoNT (no)	N
9	F	51	2	Jaw	L jaw deviation	Speech, eating	R crown replacement and repair	Tongue depressor between teeth on the L	Jaw pain	CLZ (unknown)	N
10	F	56	2	Jaw	R jaw deviation	Speech, chewing	None	Plastic between teeth on the right and in front, holding jaw	Mild pain	THP (yes, 6 mg/d)	N
11	F	58	1	Jaw	L jaw deviation	Speech, eating	3 wks after L subdural hematoma	Plastic between teeth	None	THP (unknown)	N
12	F	50	1	Jaw	L jaw deviation	Speech	None	Holding the L jaw, imagination about holding the jaw, tongue depressor/syringe between teeth	None	THP (unknown)	N
13	M	66	7	Jaw, tongue	Jaw opening and tongue mvmts	None	None	Straw in his mouth, lightly touching lips	Difficulty chewing	CLZ (no), BoNT (no), THP (unknown)	N
14	F	56	1	Jaw, tongue	Jaw opening, tongue protrusion, alternating tongue deviation	Speech -- > eating, chewing on the R side	3 mo after traumatic facial abrasion	None	Clicking in jaw	LVT (no), TBZ (no), THP (unknown)	Y; no response
15	F	44	11	Jaw, tongue	Jaw closing, tongue retraction	Speech, chewing	Ill-fitting new denture leading to painful jaw and facial swelling	None	None	CLZ (unknown), THP (no), Baclofen (unknown), BoNT (yes, mild)	N
16	M	41	3	Jaw	Jaw protrusion	At rest, speech, eating	Skull fx and extensive L orbital bone damage, R lower molar extraction 1 y later	Tongue depressor or a pen cap between teeth	None	CLZ (yes, mild, 3–4 mg/d), THP (unknown)	Y; no response
17	M	55	2	Tongue	Tongue protrusion with 90° clockwise rotation	At rest, absent during tasks	None	None	None	LD (no), CLZ (yes, mild, 3 mg/d), BoNT (no), THP (unknown)	N
18	F	78	4	Lower face	Multiple mvmts with coexisting ET	Arrhythmic facial movements (talking > at rest); rhythmic facial movements (at rest > talking)	Dental procedure to crown several lower molar teeth	Holding chin with hand	Voice straining (started after the dental procedure), facial movements started 2 years after.	TBZ (no), CLZ (no), THP (unknown)	N
19	F	67	7	Tongue	Tongue protrusion	At rest, chewing/eating	None	None	Mild drooling and sl softening of voice, jaw tremor. Mouth ulcer	TBZ (unknown)	N
20	F	63	1	Jaw	Jaw opening, jaw protrusion	Speech (words with an "r" or "l", at rest		Holding a plastic syringe in the left corner of mouth	Difficulty swallowing liquids	LD (no), LZP (no), Baclofen (unknown)	N
21	M	45	1	Jaw	Jaw closing, jaw protrusion and L jaw deviation	At rest	None	Tongue depressor between teeth on the right side, touching L lower cheek	Clicking noise at the L jaw, jaw pain (L > R)	CLZ (no), Cyclobenzaprine (yes, mild), Jaw surgery (no), THP (unknown), BoNT (unknown)	Y; no response
22	M	45	2	Jaw, tongue	Jaw opening, tongue retraction	At rest, chewing	?cocaine abuse	None	None	Clonidine (yes, mild), CBZ (mild), CLZ (unknown), THP (unknown)	N
23	M	51	1	Jaw	L jaw deviation, mild jaw protrusion	At rest	None	Firm pressure on R cheek, holding object between teeth on the L side	None	LD (no), BoNT (unknown)	N
24	F	51	15	Lip, tongue, perioral muscles	Invol. dystonic mvmt of face, lips and tongue; invol. sucking mvmt of mouth	Speech, eating	None	Tongue depressor between teeth, putting hand on face	Dysphagia, 8-lb wt loss, biting cheek-- > mouth ulcers	THP (no), CLZ (no, confusion, memory problem), BoNT (yes, mild)	N
25	M	25	25	Jaw, perioral muscles	Mouth closure with sl jaw opening and protrusion	Speech, praying, chewing	None	Tongue depressor between teeth (L > R), touching jaw/chin lightly	Pain at b/l lower jaw, burning sensation in buccal mucosa, biting buccal mucosa-- > bleeding	CLZ (no, 1.5 mg/d), THP (no, 2 mg/d, dose inadequate)	N
*26*	*M*	*50*		*Tongue*	*Tongue rolling*	*Speech*		*Toothpick in mouth; touch lips with finger*		*TBZ (no); THP (unknown)*	*Y; good response*
*27*	*M*	*38*		*Jaw*	*L jaw deviation*	*Speech, chewing*		*Pipette between left molars; touch left jaw*		*CLZ, THP (no)*	*Y; no response*
*28*	*F*	*60*		*Jaw*	*Jaw closure*	*Non-specific, worse with speech*		*Touch L cheek; plastic tube between teeth*		*BoNT (unknown)*	*N*
*29*	*F*	*43*		*Jaw*	*Jaw closure and L jaw deviation*	*Bassoon -- > speech, chewing*		*Device over molar; plastic appliance between teeth*		*BoNT (yes)*	*Prior device; good response*
*30*	*M*	*41*		*Jaw*	*Jaw closure*	*French horn -- > speech, drinking*		*Bite plate; straw between teeth on L*		*BoNT, CLZ, baclofen, THP (no to all)*	*Y; brief response*
*31*	*F*	*41*		*Jaw*	*Jaw protrusion and left jaw deviation*	*Speech*		*Straw between teeth, L > R*		*BoNT (unknown)*	*Y (bite blocks); help when bite*
*32*	*M*	*33*		*Lip*	*Lip pulling to the corners of the mouth*	*Speech*		*Straw between upper lip-teeth; hold upper lip with 2 fingers*		*THP (yes, 6–18 mg/d)*	*N*
*33*	*F*	*51*		*Platysma, face*	*Neck pull forward, grimacing*	*Non-specific*		*Straw between teeth on either side*		*CLZ (yes, 0.5 mg/d), BoNT (no)*	*N*
*34*	*M*	*64*		*Pharynx, larynx*	*Pulling*	*Paradoxical; worse at rest, speech better*		*Plastic between teeth*		*CLZ (unknown)*	*N*
*35*	*F*	*68*		*Jaw, tongue*	*Lateral movements of jaw, tongue rolling*	*Non-specific*		*Straw/spoon between teeth*		*THP (no); CLZ (yes, 1 mg/d)*	*Y; good response*
*36*	*F*	*66*		*Tongue*	*Tongue pushing against teeth (protrusion?)*	*Non-specific*		*Paper towel over lower front teeth; toothpick*		*Unknown*	*Unknown*
*37*	*M*	*48*		*Perioral muscles*	*Rotation of perioral muscles*	*Speech*		*Straw in his mouth*		*BoNT (yes)*	*Y; unknown*
*38*	*M*	*44*		*Tongue*	*Tongue rolling*	*Flute -- > speech*		*Plastic pipette in cheek*		*THP (yes, 4–6 mg/d)*	*N*
*41*	*M*	*30*		*Embouchure*	*Lowering, protrusion*	*Trombone*		*Touch chin or L side of face*		*None*	*N*
*42*	*M*	*66*		*Jaw, tongue*	*Jaw opening, tongue movements*	*Paradoxical; worse at rest, speech better*		*Straw between teeth*		*THP, CLZ, BoNT (no to all)*	*Not yet referred to M.G.*
*43*	*F*	*42*		*Jaw, lips*	*Jaw and lip opening*	*Speech, chewing*		*Touch R temple*		*CLZ, THP, BoNT, baclofen (no to all)*	*Y; brief response*

Data on our new patients (number 1–25) is presented with previously published patients (number 26–43; shown in italics).

Legend: Patient number, gender, age at onset (years), duration of the disease (years), areas that are affected (arrow shows the pattern of spreading from one region to another), primary movement, task specificity, precipitating events, sensory trick, associated symptoms, treatment employed, and trial of oral devices and response are listed in columns. Patients 1 to 25 were new and patients 26 to 43 (shown in italics) were previously published [4]. Patients 39, 40 and 44 were excluded due to co-existing blepharospasm. There is no information on precipitating event in patients 26 to 43.

M, male; F, female; L, left; R, right; mvmt, movement; invol., involuntary; >, had greater effect than; fx, fracture; wk, week; mo, month; y, year; TMJ, temporomandibular joint; sl, slight; wt, weight; b/l, bilateral; LD, levodopa; BoNT, botulinum toxin injections; THP, trihexyphenidyl; CLZ, clonazepam; mg/d, milligram per day; DZP, diazepam; LVT, levetiracetam; TBZ, tetrabenazine; N, no; Y, yes. In treatment column, response is shown in the brackets (yes, no, unknown): “yes” (in patients 1 to 25), a good response; “yes, mild”, mild response, effective dose indicated in the brackets. In the oral device column, response is shown as Y and N (yes and no).

To make description of this entity more comprehensive, we have included the 16 patients with idiopathic LCrD from our previous publication in our series [4]. We excluded three patients from the previous publication who also had blepharospasm. The study was approved by the Mount Sinai Institutional Review Board. Video written-informed consent was obtained from all patients. We did not obtain separated informed consent as this is a retrospective chart review.

## Results

Of 25 patients identified in the current series, 18 (68%) were female, and the mean age at evaluation was 60 years (range 44–82). Mean age at onset of symptoms was 56 (range, 25–78), 59.5 years in women and 48.6 in men. Only one patient had a positive family history, a patient with a son with an identical phenotype of rightward jaw deviation. The most common primary phenotype was a mixed dystonia (12 patients, 48%). Of the mixed phenotypes, jaw opening was the most common primary movement (4 patients). The second most common primary phenotype was jaw deviation (6 patients, 24%), followed by jaw opening (4 patients, 16%). Of 6 patients with pure jaw deviation, the jaw was deviated to the left in 4 patients and to the right in 2 patients. There was one patient each with pure jaw protrusion, pure tongue protrusion and pharyngeal/laryngeal dystonia.

In almost all patients (24/25, 96%), dystonia was task-specific. The most common task triggering dystonia was speaking (16/25, 64%), followed by eating or chewing (15/25, 60%). Thirteen patients had dystonia related to both speaking and eating/chewing. Four of these 13 demonstrated a pattern of progression of task-specificity, with dystonia initially involving speech and then progressing over time to involve eating as well. Some patients who had dystonia with chewing had difficulty specifically with hard objects. A few patients with dystonia related to speaking described a trigger with particular sounds, such as “r” or “l” sounds in one patient, and “ke” or “ge” sounds in another. Dystonia also occurred at rest in 9 patients.

Eleven patients (44%) reported a precipitating event before onset of dystonia, the most common of which was dental work within the preceding several weeks (5 patients, 20%). Two precipitating events related to injury or manipulation of maxillofacial bones included a traumatic mandibular fracture after a fall and maxillary bridge replacement. The latter patient was required to hold her mouth open for 2 hours while the temporary bridge was fit. Other precipitating events included a history of a left subdural hematoma 3 weeks prior, and a skull fracture with extensive left orbital bone damage from a car accident.

Geste antagonistes were found in 18 patients (72%). The most common one observed on examination was holding an object such as a piece of plastic, a tongue depressor, syringe or straw between the teeth, in 17 out of 18 patients. Six patients demonstrated marked side specificity of the geste: improvement when placing the trick device between teeth on one side but not the other side. Complete data on side specificity in all patients could not be assessed due to lack of testing. There was no clear relationship between the phenomenology of dystonia (for example, the side that the jaw deviated to) and the side specificity of this geste antagoniste. Ten patients also had improvement in dystonia when lightly touching the jaw, chin or face with finger(s) or a hand. One of these ten patients had improvement even when he imagined holding his jaw. Three reported improvement with chewing gum, one of which had side specificity on the left.

Stress and fatigue were aggravating factors in some patients. The most common associated symptom was jaw pain (5 patients, 20%). A clicking noise in the jaw or ear was found in 4 patients (16%). Dysphagia was found in 2 patients: one had severe weight loss and the other had dysphagia only with liquids. Two patients reported mouth ulcers or bleeding from biting buccal mucosa.

Oral medication and botulinum toxin injections were employed in treatment. Oral medications included trihexyphenidyl (21 patients, 84%), clonazepam (15 patients, 60%), baclofen (5 patients, 20%) and levodopa (4 patients, 16%). Propranolol, carbamazepine and clonidine were each also used in one patient. The most common daily dose of trihexyphenidyl was 6 mg/day in 10 patients (range 1–19 mg). Of 21 patients treated with trihexyphenidyl, three had response with very good benefit at the dose of 2–15 mg/day, seven did not have benefit and the response was unknown in 11 patients. Of 15 patients treated with clonazepam, two had response that was only mild at the dose of 1.5-3 mg/day, nine had no response and the response was unknown in four patients. Baclofen was employed in 5 patients, none of whom had a good response, three had no response and the response was unknown in the other two patients. Of data available, the dose of baclofen employed was 10–30 mg/day.

Ten patients (40%) underwent botulinum toxin (BoNT) injections. The sites of the injections depended on the primary movement, mostly external or internal pterygoids. There was a response that was only mild in three patients, one of whom continued to have significant disability. Five patients had no response to BoNT injections and the response was unknown in two patients.

With regards to side effects, confusion, mild tendency to sadness and anxiety each were found in one patient each taking trihexyphenidyl at 3–6 mg/day. Side effects from clonazepam included anxiety, confusion/memory problem, and sedation, each found in one patient taking the dose of 1–1.5 mg/day. One patient who received BoNT injections was complicated by marked dysphagia. Depression and rash each were found in one patient taking tetrabenazine.

Table 2 illustrates the comparison of results combining our 25 new patients with 16 patients from the previously published series, when analyzing the new patients compared to all 41 patients. The results with regards to sex predilection, areas affected, primary movement, task-specificity, geste antagoniste, treatment employed and response to the treatment were similar. Of note, 3 patients from the previously published series had task specificity related to performance on a brass or woodwind musical instrument (embouchure dystonia).

**Table 2:** **Comparison of results between new patients and combined data with the previous publication**

	New patients	Combined data
**Number of the patients**	25	41
**Sex**	Female 18/25 (72%)	Female 24/41 (58.5%)
	Male 7/25 (28%)	Male 17/41 (41.5%)
Female/male (F/M ratio)	2.6:1	1.4:1
**Average age at onset (years)**	**56**	**53.3**
In female subgroup	59.5	48.6
In male subgroup	48.6	47.2
**Areas affected**		
Jaw only (including mixed primary movements of jaw)	15 (60%)	20 (48.8%)
Lip or perioral only	0 (0%)	2 (4.9%)
Tongue only	2 (8%)	5 (12.2%)
Pharynx/larynx	1 (4%)	2 (4.9%)
Mixed	7 (28%)	11 (26.8%)
Jaw and tongue	4 (16%)	6 (14.6%)
**Primary movement**		
Pure jaw deviation	6 (24%)	7 (17%)
to the left	4 (16%)	5 (12.2%)
Pure jaw opening	4 (16%)	4 (9.8%)
Mixed	12 (48%)	17 (41.5%)
**Task specificity**		
Speech	18 (72%)	27 (65.9%)
Eating/chewing	15 (60%)	18 (43.9%)
At rest	9 (36%)	11 (26.8%)
Musical instrument	0 (0%)	4 (9.8%)
**Geste antagoniste**	**18 (72%)**	**34 (82.9%)**
Object between teeth	17 (68%)	31 (75.6%)
with side specificity	6 (24%)	8 (19.5%)
Holding body part lightly	10 (40%)	15 (36.6%)
with side specificity	4 (16%)	8 (19.5%)
**Treatment**		
**THP**	**21 (84%)**	**29 (70.7%)**
good response	3 (12%)	5 (12.2%)
**CLZ**	**15 (60%)**	**22 (53.7%)**
good response	2 (8%) - all mild	4 (9.8%)
**Baclofen**	**5 (20%)**	**7 (17%)**
good response	0 (0%)	0 (0%)
**BoNT**	**10 (40%)**	**18 (43.9%)**
good response	3 (12%) - all mild	5 (12.2%)
**Dental device**	**4 (16%)**	**11 (26.8%)**
good response	0 (0%)	2 (4.9%)

Legend: Data on our 25 patients and combined 41 is shown in the middle and right columns, respectively. Number of patients, sex, average age at onset, areas affected, primary movement, task specificity, geste antagoniste, and treatment employed are listed in the left column. With regards to areas affected, note that “jaw only” group also includes patients with mixed primary movement of the jaw such as mixed jaw opening and protrusion. With regards to geste antagoniste, data on side specificity, either left or right, is shown. Numbers of the patients on each treatment modality and the ones with a good response are shown. Data on “no-response” and “unknown” group is not shown here. All numerical data (except average age at onset) represent numbers of the patient with percentage (in the brackets) of total number of the patients in each group, 25 and 41, respectively, are shown.

THP, trihexyphenidyl; CLZ, clonazepam; BoNT, botulinum toxin injections.

The following case histories illustrate the clinical entity (see video in Additional file 1).

### Patient 2: pure jaw deviation

A 72-year-old woman suffered a traumatic mandible fracture after a fall. Her jaw was wired shut for six weeks. On recovering from surgery, she immediately became aware of a change in the way her jaw felt. She noticed that her right molars were no longer contacting one another when she chewed; eating, chewing and speaking were difficult, and her jaw would move spontaneously to the left. She had no history of neuroleptic or anti-emetic exposure. She was not aware of any sensory tricks. Examination revealed mild jaw dystonia with leftward shift, mildly accentuated by speaking. There was no clear change with holding a plastic syringe between her teeth on either side. She was started on trihexyphenidyl 2 mg daily with titration to 2 mg twice a day. She had a very good sustained response even when decreasing the dose down to 2 mg/day.

### Patient 17: pure lingual dystonia

A 57-year-old man developed involuntary movements of the tongue over a period of two years. He did not identify any sensory tricks, and there was no history of precipitating factors, neuroleptic or antiemetic exposure. Examination revealed mild protrusion and rotation of the tongue in a 90-degree clockwise fashion. Movements were not triggered by tasks, and indeed were absent when he spoke. We were not able to identify any sensory tricks such as placing a tongue depressor or a small plastic bite block between his teeth. He had been treated with botulinum toxin injections, without apparent benefit, and with clonazepam, with mild benefit at the dose of 3 mg/day. He was then started on trihexyphenidyl with a slow titration schedule up to 6 mg/day (in 3 divided doses) without either benefits or side effects. We increased the dose to 12 mg/day (in 3 divided doses). The response with this dose was unknown as further follow-up information was not available.

### Patient 3: mixed dystonia

A 61-year-old woman underwent a maxillary bridge replacement four years prior to being seen. A temporary bridge was made and her mouth was open for two hours while this was fit. Since that time, she felt that her jaw alignment was incorrect, and she developed difficulty with her speech, jaw pain and clicking in her temporomandibular joints (TMJs). In the last year she noticed movements involving the tongue and jaw, initially limited to the task of speaking. Over time, movements spread to involve eating and chewing, sparing drinking. There was no history of neuroleptic or anti-emetic exposure. She saw a dentist and tried multiple oral devices without benefit. Examination revealed a tendency of the jaw to open and push forward anteriorly at rest. When she spoke, movements were further activated causing mild dysarthria. Holding a piece of plastic between her teeth immediately improved her speaking. She was started on low dose trihexyphenidyl with slow titration to 2 mg three times daily without benefit. We then referred her for botulinum toxin injections.

## Discussion

In this series, the largest of such patients reported, idiopathic LCrD was more common in women than men (2:1). This is similar to the pre-existing literature on LCrD and other forms of focal dystonia such as cervical dystonia (female/male; F/M ratio 1.2-1.92:1) [9–17], blepharospasm (F/M ratio 1.35-2:1) [9, 18–20], spasmodic dysphonia (F/M ratio 1.35-15:1) [9, 21–24], and oromandibular dystonia 3.28:1 [9], but opposite to what is seen in writer’s cramp (F/M ratio of 1:2) [9]. Almost half of our patients had a mixed phenotype and the other half were simpler (pure). In the group with pure phenotype, the most common primary movement was jaw deviation, whereas jaw opening was the most common phenotype of mixed phenotype. Jaw protrusion, as well as tongue and pharyngeal dystonia was much less common.

The anatomy and function of the jaw is unique. Embryologically the jaw muscles are derived from presomitic mesoderm of the first branchial arch [25], whereas limb muscles are derived from somites. The multiple functions of the jaw are complex as well. Chewing requires complex coordinated movements of various jaw muscles. Masticatory myosin is expressed in the jaw, and during evolution it is replaced with other types of myosin to tailor for eating habits or types of diet [26]. In humans jaw closing muscles contain rich muscle spindle innervation whereas jaw-opening muscles do not [27, 28], possibly due to the strong proprioceptive input related to jaw closure for modifying bite strength. One possible reason for this is the evolutionary pressure that modifies the bite. For example, humans can modulate bite strength to match different consistencies of food, and many mammals and vertebrates must be able to exert tremendous force with the jaw to capture prey, but also modify their bite so as to gently carry their young. These unique features of the jaw muscles might explain the common and often robust sensory gestes observed in our patients.

Task specificity is a nearly universal feature of LCrD, and the most common triggering tasks were speaking, followed by eating or chewing. Often dystonia began with speaking, and then spread to eating or chewing. We did not observe other patterns of spread of dystonic tasks. Recent dental work or oral trauma may precipitate LCrD, as can manipulation of the facial bones, especially the maxilla and mandible. There may be a role of “osseoperception” [29, 30]; afferent signal from periodontal mechanoreceptor is required in fine motor control of the mandible such as chewing or jaw closing. However, this concept can explain only some, but not all, patients such as in the ones who had tooth extraction as a precipitating event. In addition, it does not explain non-jaw related dystonia such as lingual dystonia after dental work.

3/4 of our patients possessed a geste antagoniste, and all of those had improvement of LCrD by holding an object between their teeth. Some improved when lightly holding the chin and jaw, and occasionally with imagination of the sensory trick. This feature is a key diagnostic aide, and the presence or absence of a sensory trick should be investigated in all such patients. The numbers are too small to support a clear relationship between the presence of a geste antagoniste and treatment response in our series. Of 9 patients with response to at least one treatment modality, six had at least one sensory trick, whereas 12 of 16 patients without treatment response did not have an identified sensory trick.

The most common treatment used in our patients was trihexyphenidyl, the response of which was more robust than the other modalities, but still disappointing. None of the patients had good response to baclofen or dental device in our series. Response to botulinum toxin injection was less robust than in other series, for reasons that are not clear.

## Conclusion

Idiopathic LCrD is often missed and institution of effective therapy is often delayed. The clinical features and natural history of LCrD are similar to other forms of focal dystonia.

We offer the following practical guidelines for clinicians who evaluate patients with LCrD. Table 3 illustrates clinical approach to patients with LCrD. The differential diagnoses of idiopathic LCrD includes other primary dystonia (such as primary segmental dystonia, Meige’s syndrome), secondary dystonia (such as tardive dystonia from dopamine receptor blocking agents, infectious dystonia including anti-NMDA encephalitis), heredodegenerative dystonia (such as X-linked dystonia parkinsonism or Lubag’s disease, neuroacanthocytosis, Lesch-Nyhan syndrome, SCA 8 [31], cerebrotendinous xanthomatosis) [32], and pseudodystonia. Pseudodystonia in the differential diagnoses of LCrD include Isaac’s syndrome, tetanus, and musculoskeletal abnormalities such as Satoyoshi syndrome.

**Table 3:** **Practical guideline in evaluation of patients with LCrD**

History	
•	Onset
•	Description of the abnormal movement or sensation
•	Precipitating factor (especially history of recent dental work or maxillofacial trauma) lower cranial
•	Aggravating and relieving factors
•	Associated symptoms such as pain, jaw clicking
•	Sensory tricks
•	History of previous treatment such as dental prosthesis
•	History of secondary causes of dystonia especially dopamine receptor blocking agent exposure
**Examination**	
•	Identify the primary movement(s)
	o Jaw in each axis: opening/closing, lateral deviation (left/right), protrusion/retraction
	o Tongue: protrusion/retraction, torsion
•	Determine task specificity (dystonia occurs with speaking, eating/chewing and/or at rest)
•	Identify sensory tricks: light touch, placing objects such as plastic syringe or tongue depressor between teeth on each side and in the center
•	Assess evidence of dystonia in other body parts especially in upper cranial region, voice and neck

Legend: Practical guideline in history taking and physical examination of patients with LCrD is described.

We hope that this paper will call attention to this entity, and aide dental professionals, general physicians and neurologists in securing the correct diagnosis.

## Electronic supplementary material

Additional file 1: **Patient 2 demonstrates pure left jaw deviation, accentuated by speaking.** **Patient 3** demonstrates jaw-opening dystonia and mild jaw protrusion, more prominent when speaking. Dystonia improved when a plastic syringe is placed between her teeth. **Patient 23** has prominent left jaw deviation and mild jaw protrusion at rest. Dystonia improves when he places a plastic syringe between his teeth on the left, but not on the right, and holding firm pressure on the right cheek. **Patient 1** demonstrates jaw opening dystonia when chewing. **Patient 4** has jaw opening dystonia, worse when speaking and chewing. Whispering and singing are easier for her. Dystonia was mildly improved when holding a lower jaw. Dystonia and speech were markedly improved when placing a plastic syringe or a tongue depressor between her teeth. **Patient 5** shows severe jaw opening dystonia, triggered when she speaks. Dystonia is improved when holding bilateral jaw with her hands or an examiner’s hands. **Patient 10** shows pure right jaw deviation, accentuated by speaking. Dystonia is improved when holding her lower jaw with a finger, and placing a plastic syringe between her teeth on the right and in front, but not on the left. **Patient 25** has mouth closure with slight jaw opening and protrusion when speaking, worse when praying. Dystonia is better when placing a tongue depressor between his teeth. Dystonia was also accentuated by chewing but to a lesser degree than speaking. (M4V 17 MB)

## Abbreviations

BoNT: Botulinum toxin
F/M: Female/male
LCrD: Lower cranial dystonia
NBIA: Neurodegeneration with brain iron accumulation
NMDA: N-methyl-D-aspartate
SCA: Spinocerebellar ataxia
TMJ: Temporomandibular joint.
